# Neurology of Androgens and Androgenic Supplements

**DOI:** 10.1007/s11910-025-01426-6

**Published:** 2025-06-03

**Authors:** Chetna Dengri, Whitney Mayberry, Ahmed Koriesh, Amre Nouh

**Affiliations:** 1 Neuroscience Institute, Cleveland Clinic Florida, 2950 Cleveland Clinic Blvd, Weston, FL 33331 USA; 2 Cleveland Clinic Florida Tradition Hospital, Port St. Lucie, FL USA

**Keywords:** Androgen, Dihydrotestosterone, Testosterone Replacement Therapy, Neuroplasticity, Neuroprotection

## Abstract

**Purpose of Review:**

This article explores the intricate relationship between androgens, androgen receptors, and the central nervous system. We examine the role of physiologically derived androgens and androgenic supplements in neurodevelopment and neuroplasticity and delve into the involvement of androgen pathways in the pathogenesis of various neurological disorders.

**Recent Findings:**

This review highlights the increasing recognition of testosterone and androgen signaling in various neurological conditions, with evidence of both protective and harmful effects depending on dosage and context. Although limited to experimental use, testosterone replacement therapy (TRT) may serve potential benefits in the management of multiple sclerosis, epilepsy, headache, Duchenne muscular dystrophy, amyotrophic lateral sclerosis, and Parkinson disease. On the other hand, androgen-blocking treatments may help alter disease progression in spinal and bulbar muscular atrophy. Testosterone supplementation can have potential adverse events when used at a supratherapeutic level, and prenatal testosterone exposure is believed to contribute to the pathogenesis of neurodevelopmental disease. Additionally, androgen-blocking agents could increase the risk of neurodegenerative conditions, such as Parkinson disease and Alzheimer disease.

**Summary:**

Despite the above findings, there is no established indication of TRT or androgen-blocking medication in neurological disorders. The body of evidence highlighting the involvement of androgens and androgen receptors (ARs) in pathogenesis of neurological diseases is growing. This includes ongoing research exploring the potential therapeutic targets involving the androgen signaling pathway for management of neurological disorders. Future placebo-controlled clinical trials are essential to determine the efficacy and safety of TRT or androgen-blocking therapies in managing neurological disease.

## Introduction

Androgens are physiologically found in both men and women but differ in quantity and function amongst the genders. The primary function of androgens involves reproduction and the development of secondary sexual characters. There has been growing interest in the role of androgens within the central nervous system (CNS), particularly considering the observed patterns in certain neurological disorders—where some show a higher prevalence in males, while others appear to manifest with milder severity and have lower prevalence in men. In this article, we discuss the different forms of endogenous androgen, their function in the CNS, the evolving understanding of the role of androgen in various CNS disorders, and the therapeutic use of androgen supplementation for CNS pathologies.

### Physiological Role of Androgen in the Central Nervous System

#### Endogenous Androgens and their Functions

The adrenal glands and gonads (testis in men and ovaries in women) produce five main forms of androgens: testosterone, dehydroepiandrosterone sulfate (DHEAS), dehydroepiandrosterone (DHEA), androstenedione, and androstenediol. DHEAS is a weak androgen, produced in the adrenal glands that act as a DHEA reservoir. Another weak androgen is DHEA, produced from DHEAS in the adrenal glands, brain, and gonads. Androstenedione has moderate androgenic activity, is produced by adrenal glands and gonads, and is derived from DHEA. Androstenedione acts as the precursor for both testosterone and estrogen. Androstenediol is an androgen that is converted into testosterone and estrogen in peripheral tissue. Testosterone is the most potent androgen, produced primarily by the Leydig cells in the testis. This androgen is responsible for masculine features and fertility in males while having positive effects on bone density, lean mass, mood, and libido in females. Testosterone is converted into dihydrotestosterone (DHT) by the action of 5-alpha reductase in the prostate and skin. It is responsible for formation of external male genitalia in fetus, prostate growth, and plays a role in male pattern baldness. In men, a small amount of testosterone is converted into estradiol in adipose tissue, bones, and brain. In women, a minute amount of testosterone is produced following peripheral conversion of DHEA and androstenedione in the liver, skin, muscles, and fat tissue. These hormones not only play an important role in the development of secondary sexual characteristics and fertility but are increasingly recognized for their role in the development and function of the CNS.

#### Role of Androgen in the Development of Nervous System

**Fig. 1:**
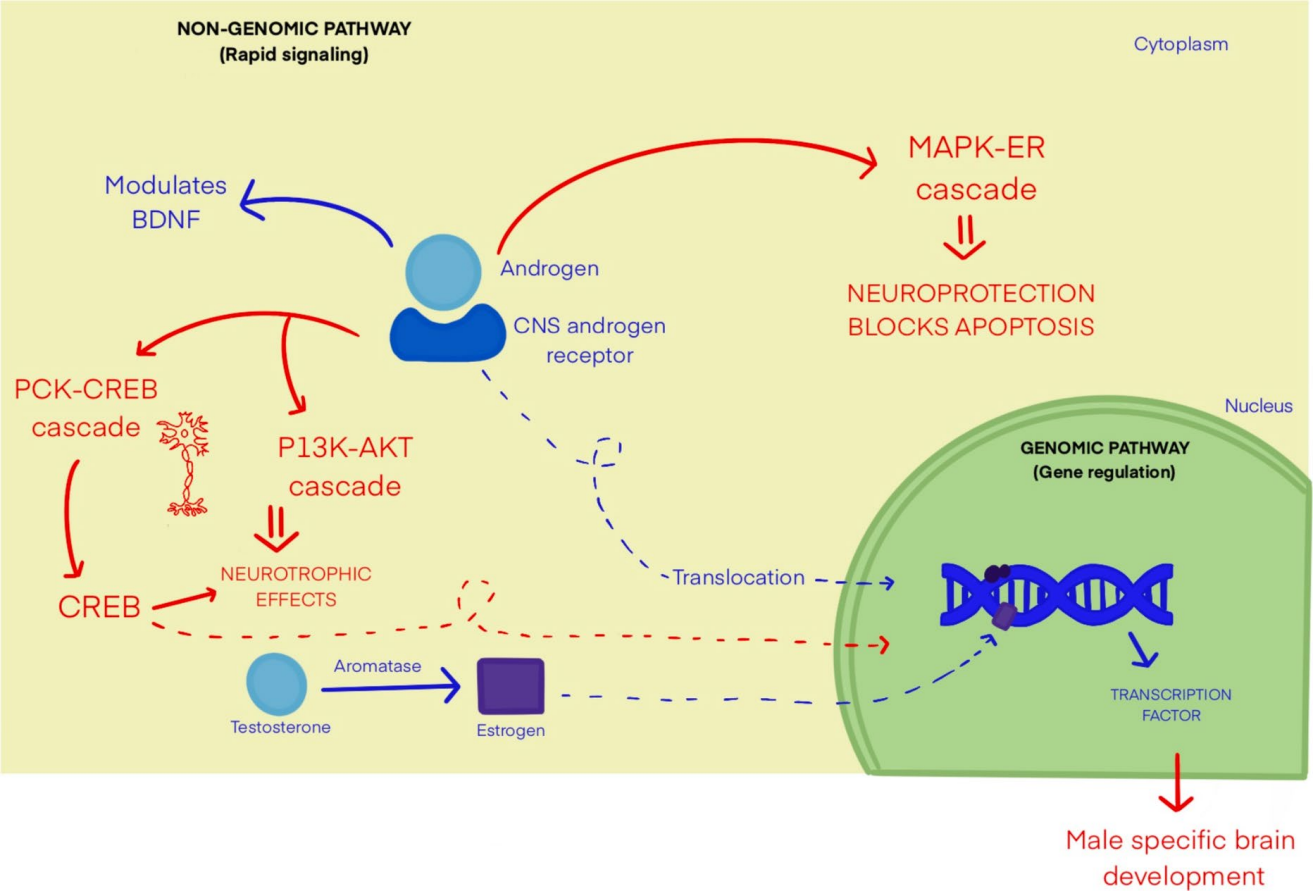
Non-genomic and genomic androgen signaling in CNS. *Legend: This figure is a simplified representation of the various androgen signaling pathways in the central nervous system. Abbreviations: BDNF: Brain-Derived Neurotrophic Factor, MAPK-ERK: Mitogen Activated Protein Kinase—Extracellular Signal-Regulated Kinase, PKC-CREB: Protein Kinase C—cAMP Response Element-Binding Protein, PI3 K-AKT: Phosphatidylinositol 3-kinase-Protein kinase B*

Androgen receptors (ARs) are expressed through a gene located on chromosome X and remain cytoplasmic in the inactive state. Following androgen binding, they convert to a nuclear receptor which influences gene expression through binding at specific DNA sequences. Androgens bind to these receptors and operate via genomic (DNA binding) or nongenomic pathways (non-DNA binding) that influence multiple signaling cascades essential for CNS function and neuroprotection. Figure 1 provides a simplified representation of androgen signaling pathways in the CNS. Although the exact location and function of ARs in the adult brain remain under investigation, animal models have demonstrated the presence of ARs at multiple CNS locations. Notably, ARs have been identified in the forebrain, thalamus, hypothalamus, amygdala, hippocampus, and olfactory bulb. ARs in cerebral locations are thought to be involved in regulation of cognitive behavior and neuroplasticity. ARs are also found in the dorsal horn of the spinal cord and various brain stem locations, predominating in the area postrema, motor nucleus of the vagus nerve, dorsal raphe nucleus, periaqueductal gray, retrorubral nucleus, retrotrapezoid nucleus, and substantia nigra. While the specific functions of ARs in these regions are still being studied, these structures are known to contribute to a broad range of processes, including motor, autonomic, and sensory functions. Thus, it is hypothesized that alterations of ARs or androgen interactions with ARs located in the CNS may play a role in various neurological diseases and serve as a target for disease management. [1–3]

The influence of androgens on brain development may begin during fetal development. Higher levels of testosterone in male fetuses are associated with decreased cortical thickness of the prefrontal cortex and are thought to have a negative influence on the connection between the cerebral cortex and the limbic system. The prefrontal cortex is responsible for executive functioning including impulse control, emotion, self-awareness, and social cognition. The associated connection with the limbic system influences the processing of memory and emotion. Thus, it is hypothesized that androgens negatively impact cognitive functions regulated through these structures [4]. Research has indicated individuals with autism spectrum disorder (ASD) have elevated androgen levels when compared to their peers. However, to date, research has not demonstrated a causal relationship between these elevations and development of ASD [5]. Elevations in prenatal testosterone have additionally demonstrated an inverse relationship with the development of pathways responsible for social communication and cognition [6, 7]. This is postulated to contribute to the higher incidence of certain neurodevelopmental disorders as well as increased aggressive behaviors and diminished executive functioning in males with ASD as compared to females. Research shows that fetuses exposed to elevated in-utero androgen levels and born to mothers with associated conditions—namely polycystic ovary syndrome—experience a higher rate of ASD [8]. Conversely, DHEA has the opposite effect than testosterone on brain development, possibly counteracting the effects of testosterone. DHEA leads to increased cortical thickness and has positive effects on areas of visual attention and working memory [4]. A deeper understanding of the complex relationship between testosterone, DHEA, and neurodevelopment is essential to determining clinical applications.

### Role of Androgens in Neuroplasticity

The relationship between androgens and brain development highlights the need to understand their role in neuroplasticity. Neuroplasticity is the ability of the brain to adapt in response to stimuli and is of distinct interest in stroke rehabilitation and cognitive recovery [9]. Androgens are thought to have both positive and negative impacts on neuroplasticity. Testosterone promotes the formation of new networks in the hippocampus and allows for synapse plasticity in mice models. Additionally, it has been shown to increase neurogenic output of excitatory progenitors in human brain organoids [10–12]. The ability of androgens to facilitate formation, growth, and modulation of neural networks may represent a target for neural recovery following an insult to the CNS. However, the impact of androgens on oxidative stress as well as the negative modulation of neurotrophins growth factors may have counterproductive detrimental effects [12, 13]. A deeper understanding of the mechanisms involved in neuroplasticity could guide therapeutic interventions with androgens such as testosterone replacement therapy (TRT) in neurological recovery in neurodegenerative diseases. The following section highlights our current understanding of the role of androgens in certain CNS disorders and their potential therapeutic role across neurological domains.

### Role of Androgens in Diseases of the Nervous System

#### Cerebrovascular System

The higher incidence of ischemic stroke in men, especially with hypogonadism, as well as in post-menopausal women suggests involvement of sex hormones in the pathogenesis of ischemic stroke. Dose-dependent effects of testosterone and association with ischemic stroke have been established. Testosterone at therapeutic level leads to protective effects against ischemic stroke and cardiovascular events. However, there are no guidelines advocating the use of TRT in men with hypogonadism for stroke prevention. The American Urological Association and Endocrine Society recommends TRT use only for management of symptomatic hypogonadism in men [14, 15]. Conversely, supratherapeutic TRT use as seen for cosmetic and gender affirmation purposes may be associated with increased risk of ischemic stroke, thought to be due to exaggerated endothelial injury, derangement of lipid profiles, hypercoagulability, and elevated baseline blood pressure. Supraphysiologic TRT use is being evaluated as a risk factor when evaluating young patients with Embolic Stroke of Undetermined Source (ESUS) [16].

Androgens additionally inhibit pathways involved in homocysteine metabolism, and exogenous use can result in elevated homocysteine levels. Hyperhomocysteinemia has been observed in women with polycystic ovary syndrome, who are known to have elevated testosterone levels. Elevated levels of homocysteine are responsible for accelerated atherosclerotic plaques due to oxidative stress, endothelial injury, and increased thrombosis [17]. Thus, the direct relation between androgen level and homocysteine may act as a potential mechanism for increased cardiovascular events through accelerated atherosclerosis and thromboembolism [18, 19].

The specific relationship between androgens and hemorrhagic stroke remains under-investigated. One study highlighted an association between decreased free testosterone levels and an increased risk of aneurysmal subarachnoid hemorrhage (SAH) in women [20]. However, further research is necessary to understand the mechanisms by which androgens influence SAH and to explore the potential role of associated therapies in SAH prevention.

#### Neuromuscular Disorders

Some neuromuscular conditions exhibit a higher prevalence in men, suggesting a potential pathological role of sex hormones [21].

Spinal and bulbar muscular atrophy (SBMA), also known as Kennedy disease, is an X-linked neuromuscular disease characterized by loss of lower motor neurons located in the brainstem and spinal cord. SBMA is caused by CAG expansion at the first exon of the androgen receptor gene. It fully manifests in men, typically in their third to fifth decades of life, while women with homozygous mutation have a subclinical disease course, indicating a role of androgen in pathogenesis as opposed to solely the mutant AR [22]. This is supported by animal models with mutant ARs which demonstrated neuromuscular weakness only when exposed to androgens—while androgen deprivation prevented disease development—highlighting the critical role of androgen interaction with mutant receptors in disease expression [23, 24]. Therefore, studies have explored androgen antagonists as a potential therapeutic strategy to modify disease progression. Thus far, trials have demonstrated mixed results with no statistically significant difference in progression of SBMA following exposure to a 5-alpha reductase inhibitor, while other studies demonstrated delayed progression after exposure to gonadotropin releasing hormone (GnRH) agonist in men with SBMA in comparison to controls [25–27].

Amyotrophic Lateral Sclerosis (ALS) has a 20% higher incidence in men than women. It is hypothesized that loss of function of ARs located at spinal motor neurons, skeletal muscles, and certain cranial nerves increases axonal vulnerability to various insults, contributing to disease pathogenesis [28]. Cranial nerves III, IV, and VI are notably spared in ALS, which is intriguing given their lack of ARs [29]. These observations further support the link between AR dysfunction and pathogenesis of ALS. Superoxide dismutase 1 (SOD 1) gene mutation is strongly linked to both familial and sporadic forms of ALS. A study reviewing the effects of AR antagonism in presymptomatic SOD1- G93A male mice, noted an earlier onset of myofiber atrophy when compared with female mice. Another study found that androgen supplementation led to muscle growth but worsened motor neuron death and survival. However, experiments with neural AR deletion or overexpression in SOD1-G93 A mice showed no significant impact on disease progression. Overall, the connection between androgens, ARs, and ALS remains complex and unclear, with evidence suggesting that sex-based differences might play a role [30–32].

Duchenne Muscular Dystrophy (DMD) is an X-linked recessive disorder causing progressive neuromuscular weakness primarily affecting males, typically manifesting in prepubertal boys. A study of the effects of pubertal induction with monthly testosterone injections in young boys aged 12 to 17 years receiving glucocorticoids demonstrated no effects on bone density or bone age advancement but improved muscle strength. The rapid functional decline observed in the prepubertal stage along with improved outcomes following pubertal induction speaks for possible anabolic effects of androgen on muscle strength and protein synthesis or involvement of androgen-AR interaction in DMD pathogenesis [33, 34]. Preclinical models have demonstrated decreased adipose infiltration in DMD muscles and improved muscle function in female mice treated with oral selective AR modulators [35]. Glucocorticoids commonly used in DMD improve muscle and cardiovascular strength but can lead to bone fragility and delayed puberty. Use of anabolic steroids in boys with DMD led to significant improvement in quantitative muscle tests, objective tests for muscle strength, in comparison to placebo groups [36].

Inclusion body myositis (IBM) is a progressive inflammatory myositis and use of anabolic steroids in patients with IBM led to slight improvement in muscle strength when compared to placebo [37]. Furthermore, testosterone supplementation along with exercise in patients with IBM led to an additional decrease in inflammatory response when compared to exercise alone [38]. These findings suggest that TRT or anabolic steroids may offer a promising therapeutic alternative, potentially enhancing muscle and cardiovascular function in patients with neuromuscular weakness while reducing the side effects associated with prolonged corticosteroid use.

Myotonic dystrophy type 1 (DM1) is a hereditary neuromuscular disorder associated with multiple endocrine dysfunctions. Progressive testicular atrophy causing oligospermia is seen in 80% of men with DM1 along with reduced adrenal androgen synthesis [39]. The cause for testicular and adrenal involvement remains unexplored. However, this androgen deficiency is not associated with accelerated mass loss [40]. A randomized, controlled, double-blind trial conducted in 1989 studied the effects of TRT in 40 men with myotonic dystrophy and ultimately demonstrated increased muscle mass but without positive impact on overall strength [41]. As of March 2025, there are no ongoing clinical trials investigating TRT in patients with myotonic dystrophy, likely due to a limited patient population.

#### Cognition and Behavior

Testosterone is postulated to have a protective effect against the development of dementia, as evidenced by the higher incidence of Alzheimer disease (AD) in women, who make up two-thirds of AD patients. Additionally, there is an inverse relation between serum or brain testosterone level and hippocampal volume. Numerous observational studies have linked anti-androgen therapy, commonly used in prostate cancer, with an elevated risk of AD and other neurodegenerative diseases, like Parkinson disease [42]. An epidemiological study evaluating the causal relationship between androgen and AD noted a high risk of AD with low androgen levels in men, while no effects were observed in women [43–45]. This was further confirmed by male animal models having higher amyloid beta protein deposition and lower hippocampal volume following castration when compared with the control group [46]. This points to a possible link between androgens and amyloid beta pathway and a possible neuroprotective effect through downregulating the amyloid beta toxicity. However, this link remains inconclusive. One study showed no difference in cognitive performance in hypogonadal men with mild cognitive impairment on TRT compared to those on placebo at 12 weeks follow-up. Similarly, another study with a one-year follow-up reviewed the impact of TRT versus placebo in men with MCI and symptomatic hypogonadism and showed no improvement in cognitive function [47, 48]. Thus, there is no clear role for TRT in the prevention or treatment of MCI or dementia.

Post-menopausal women account for 60% of patients with AD, with female gender being an independent risk factor for development of AD. However, it remains unclear whether the higher incidence of AD in women is primarily due to their longer life expectancy or the influence of sex hormones in the development and function of cognitive areas, particularly postulating neuroprotective effects of androgen and estrogen. Meta-analysis studying the effects of menopausal hormonal therapy found improvement in overall cognitive function after estrogen-only therapy and decline in cognitive scores with estrogen-progesterone therapy when compared to controls [49, 50]. Although considerable attempts have been made to assess the effects of TRT in men and MCI, there is a notable lack of research on the role of androgens in the development of neurodegenerative disease in women or comparing these effects across genders.

#### Movement Disorders

Parkinson disease (PD), is observed twice as frequently in men than women, typically affecting males in their fifth or sixth decade of life [51]. Additionally, men have an earlier onset of PD. Exposure to anti-androgen therapy for treatment of prostate cancer was associated with increased incidence of neurodegenerative disease like PD, and men with PD have a higher prevalence of hypogonadism compared to age-matched controls [52]. This male predominance, particularly among those with testosterone deficiency, has sparked research into the potential role of androgens in PD pathogenesis and as a therapeutic target. These considerations conflict with an open label trial that found no statistical difference in the improvement of both motor and non-motor symptoms of PD following use of TRT in men with concomitant PD and hypogonadism. The study was limited by a small sample size and the lack of long-term follow-up, which may have lacked evidence surrounding any delayed effects of TRT [53]. Conversely, a retrospective analysis of five men with PD and testosterone deficiency did show significant improvement of refractory non-motor PD symptoms following TRT [54]. Finally, additional movement disorders are noted with concomitant hypogonadism, such as ataxia, dystonia, and tremor. However, the precise role of androgens in the pathogenesis of these disorders and their potential use in treatment remains largely unexplored [55].

#### Neuroimmunology

In recent years, significant advances have been made surrounding the pathophysiology of disorders of demyelination such as multiple sclerosis (MS), neuromyelitis optica spectrum disorder (NMOSD), myelin oligodendrocyte glycoprotein antibody disease (MOGAD), and transverse myelitis. These disorders are more common in women [56]. Longitudinal clinical trials studying the effects of TRT in men with relapsing–remitting MS (RRMS) have shown neuroprotective effects via immunomodulation as evidenced by improved cognitive function, slower brain atrophy, and increased production of growth factors [57, 58]. This data highlights the potential protective effects of androgens in demyelinating disorders. To further assess this concept, testosterone treatment was initiated in mice models exposed to toxins causing damage to oligodendrocytes. The study demonstrated promising results with reversal of myelin damage and stimulation of myelin formation following testosterone use in mice that had neural AR. Thus, highlighting ARs as a potential target for therapies of remyelination [59, 60]. Further clinical trials studying the effect of TRT on gray matter volume in patients with RRMS reinforced the benefit of TRT-induced remyelination by demonstrating arrest of gray matter loss when exposed to testosterone [61]. Larger placebo-controlled trials are essential to establish the safety and efficacy of TRT in patients with RRMS along with expanding research to include patients with NMO, MOGAD, and other related disorders. TOTEM-RRMS is an ongoing phase II, multicenter, placebo-controlled, double-blind trial studying MS progression in testosterone deficient men with TRT [62]. Future studies should deepen our understanding of TRTs’ effects on MS in men with testosterone deficiency and those with normal levels along with optimizing therapeutic strategies across a broader spectrum of demyelinating diseases.

#### Epilepsy Disorders

Androgens have antiseizure effects, which are further augmented when used with an aromatase inhibitor that decreases the conversion of androgen into the proconvulsant estradiol and increases levels of androgen [63]. These effects are also observed in women with catamenial epilepsy who experience decreased seizure frequency during the follicular phase of the menstrual cycle and improved seizure control in men who received testosterone supplements [64, 65]. The mechanism behind the protective effects of androgen on epilepsy remains unclear. There is no evidence supporting the role of TRT for management of epilepsy. Placebo-controlled trials are necessary to ascertain the role and safety of TRT in men with epilepsy. Some antiepileptic drugs, namely phenytoin, phenobarbital, carbamazepine, oxcarbazepine, and eslicarbazepine, are known to decrease free testosterone androgen levels in males and can cause potential side effects due to hypogonadism [66]. Studies have shown that epileptiform discharges, specifically from the temporal lobe, can influence the hypothalamic-pituitary-adrenal axis and produce symptoms secondary to sex-hormone imbalance. This needs to be further studied to understand if the pattern of hormonal imbalance can aid in seizure localization [67].

#### Headache Medicine

The role of sex hormones in headache medicine is an emerging area of interest, though current literature largely focuses on female hormones and their association with migraines. More recently, attention has shifted toward understanding the role of androgens, with growing evidence suggesting testosterone may influence pathogenesis and modulate symptom severity and frequency of primary headache disorders. Observational studies have found low testosterone and elevated estrogen levels in men with chronic migraines, as well as more pronounced symptoms of androgen deficiency and decreased severity of migraine headache in small cohorts of premenopausal and postmenopausal females following TRT use [68–70]. Potential mechanisms for the antinociceptive effect of androgens are thought to be due to their inhibition of cortical spreading depression, inhibition of estrogen and NMDA (N-methyl-D-aspartate) receptor activity, increased serotonin synthesis, and increased GABA (Gamma-aminobutyric acid) signaling. There is additional consideration of associated modulation of neuroinflammation via pathways outside CGRP (Calcitonin gene-related peptide) mediated mechanisms [71]. Cluster headaches have male prevalence and are associated with hypogonadism; the use of TRT has demonstrated promising results for refractory cases, especially in men with hypogonadism [72, 73]. Further prospective clinical trials are warranted to understand safety of TRT and identify patient population that will benefit from TRT use for management of their primary headache disorders.

Table 1 summaries the role of androgen in various neurological disorders.

**Table 1:** Summary of the Role of Androgens in Neurological Disorders

	Role of Androgen in Disease Pathogenesis	Evidence
Cerebrovascular	Ischemic Stroke: - Higher incidence in men with hypogonadism and postmenopausal females Hemorrhagic Stroke - Possible protective effects of androgen but mechanisms unexplored	- Testosterone at supratherapeutic level is associated with higher incidence of ischemic stroke [16] - Decreased free testosterone levels are associated with increased risk of aneurysmal SAH in women [20]
Neuromuscular System	Higher prevalence in men Spinal Bulbar and Muscular Atrophy: - X-linked, CAG expansion on the first exon of the androgen receptor gene - Disease pathology thought to be secondary to interaction between androgen with the mutant AR Amyotrophic Lateral Sclerosis: - Loss of function of androgen receptors at specific CNS locations is thought to increase axonal vulnerability to various insults Duchenne Muscular Dystrophy: - X-linked recessive; common in prepubertal boys Inclusion Body Myositis: - Unexplored role in pathogenesis Myotonic Dystrophy Type 1: - Progressive testicular atrophy seen in 80% men with DM1	- Androgen deprivation in mice models for SBMA with mutant gene was not associated with development of weakness [23, 24] - Women with homozygous mutation have subclinical disease course of SBMA. [22] - Delayed progression after exposure to GnRH agonist in men with SBMA in comparison to controls [27] - No statistically significant difference in progression of SBMA following exposure to a 5-alpha reductase inhibitor [25] - AR antagonists in presymptomatic SOD1- G93 A male mice had earlier onset of myofiber atrophy when compared with female mice [30] - Androgen supplementation led to muscle growth but worsened motor neuron death and survival [31] - Neural AR deletion or overexpression in SOD1-G93 A mice showed no significant impact on disease progression [32] - Pubertal induction in prepubertal boys with DMD leads to improved muscle strength [33, 34] - Decreased adipose infiltration in DMD muscles and improved muscle function in female mice treated with oral selective AR receptor modulators [35] - Anabolic steroids in boys with DMD led to significant improvement in quantitative muscle tests [36] - Use of anabolic steroids or TRT in patients with IBM led to improved muscle strength and slightly decreased inflammatory response respectively when compared to placebo [38] - Randomized, controlled, double-blind trial studying the effects of TRT in 40 men with myotonic dystrophy demonstrated increased muscle mass without positive impact on overall strength [41]
Cognitive and Behavioral	Alzheimer Disease: - Female predominant; mostly postmenopausal - Androgens are protective - Exposure to anti-androgen therapy is associated with higher incidence of AD	- Inverse relationship between serum and brain testosterone level with hippocampal volume [42] - Higher amyloid beta deposition and low hippocampal volume in castrated mice [45] - No difference in cognitive performance in men with MCI and hypogonadism following treatment with TRT at 12-week follow-up in one study and 1-year follow-up in another [47, 48]
Movement Disorder	Parkinson Disease: - Male predominant - Exposure to antiandrogen therapy is associated with increased incidence of PD	- Higher prevalence of hypogonadism in men with PD [52] - An open label trial showed no significant improvement of motor and non-motor symptoms in men with PD and hypogonadism after TRT use [53] - A retrospective analysis of 5 patients with PD and hypogonadism showed improvement in refractory non-motor symptoms following TRT use in men [54]
Neuroimmunology	Multiple Sclerosis: - Female predominant - Androgens are thought to be neuroprotective via immunomodulation No data on the role of androgen in MOGAD, NMO, Transverse Myelitis	- Men with RRMS and TRT have improved cognitive function, slower brain atrophy, and increased production of growth factors [57, 58] - Arrest of gray matter loss following TRT use in patients with RRMS [61]
Headache Medicine	Migraine Headache: - Female predominance - Androgen exert antinociceptive effects via inhibition of cortical spreading depression, NMDA receptor activity, increased serotonin synthesis, and GABA, modulation of neuroinflammation (CGRP and non-CGRP mechanisms) Cluster Headache: - Male predominant - Androgen mediated antinociceptive effect through modulation of neuroinflammation pathway and possible hypothalamic dysfunction	- Low testosterone in men with chronic migraine [68] - More pronounced symptoms of androgen deficiency in men with migraine [69] - Decreased migraine headache severity in women receiving TRT [70] - Compensated hypogonadism seen in men with cluster headaches [72] - Headache remission following TRT use in patients with low testosterone level and refractory cluster headache [73]
Epilepsy	Androgens have antiseizure effects	- Improved seizure control in men receiving TRT [63] - Decreased seizure frequency in women with catamenial epilepsy during follicular phase [64] - Decreased seizure frequency following use of TRT and aromatase inhibitor [65]

### Current Trends of Androgen Supplementation, Safety, and Indications

Androgen supplements are herbal or pharmacological in nature. The common pharmacological supplements are either DHEA or TRT, with TRT being the most widely recognized. Immense growth is underway in the global TRT market with a projected increase in dollars spent of 700 million (USD) in the coming decade [74]. This is attributed to the increased access to hormonal supplements via online shops, increased awareness around presentation of hypogonadism, and marketing of TRT for cosmetic indications or as an anti-aging supplement. This also increases the potential risks of unregulated use. Additionally, TRT has gained popularity for gender affirmation. The safety of TRT has only been established for management of symptomatic hypogonadism and is recommended by the American Urological Association and Endocrine Society guidelines. However, many alternative uses lack evidence-based understanding of associated risks.

TRT is available in multiple forms including injectables, oral formulations, subcutaneous implants, topical gels, and transdermal patches. Each delivery method differs in pharmacokinetics and side effect profiles. The cardiovascular adverse effect profile depends on the degree of hormonal fluctuation and the impact on hematocrit, lipid panel, and blood pressure. Injectable testosterone tends to cause significant fluctuations in serum levels and is associated with the highest cardiovascular risk, followed by oral formulations. In contrast, transdermal options—such as gels and patches—provide more stable hormone levels and are linked to the lowest cardiovascular risk. In 2015, the U.S. Food and Drug Administration issued a black box warning for TRT due to the increased risk of cardiovascular events, like ischemic stroke or myocardial infarction [75]. Patients on TRT are advised to undergo close monitoring to assess for polycythemia, arterial hypertension, and deranged lipid panels to mitigate these potential cardiovascular events. Additionally, there are no specific guidelines from the American Heart Association regarding the management of TRT following a cardiovascular event or use of TRT in patients with recent cardiovascular events. It is advised to do an individualized risk assessment along with shared decision making with each patient [76, 77]. Table 2 summarises the various TRT preparations and their side effects.

**Table 2:** Types of Testosterone Replacement Therapy

TRT Preparation	Route of Administration	Common Side Effects	Preparation-specific Side Effects	Potential for Cardiovascular Side Effects
Pills	Oral	***Short term:*** allergic reaction, acne, fluid retention, mood swings, hair loss, exacerbation of benign prostatic hyperplasia symptoms, hypertension ***Long term:*** Gonadal atrophy, hepatotoxicity, depression, suicidal thought, erythrocytosis, increased low-density lipoprotein, decreased high-density lipoprotein ***Potential Cardiovascular risks:*** pulmonary embolism, atrial fibrillation, ischemic stroke, and myocardial infarction in specific groups	Hepatotoxicity	Moderate
Injectable	Intramuscular		Injection site reaction, micro embolism of ester, fluctuating testosterone level	High
Patch	Transdermal		Skin irritation	Low
Gel	Transdermal			Low
Implants	Subcutaneous		Infection, pellet extrusion	Moderate

It is worth noting that clinical guidelines surrounding the use of TRT focus primarily on its cardiovascular safety and benefit. However, there is a notable absence of comprehensive data regarding the benefit and safety outside of the cardiovascular system. At present, there are no established guidelines recommending the use of TRT for the treatment of neurological conditions. Ongoing clinical trials are investigating the use of TRT in the context of neurological conditions as outlined in the preceding sections of this review.

## Conclusion

In conclusion, various neurological disorders exhibit male predominance, while some demonstrate reduced disease severity in men. Emerging evidence from preclinical models, observational studies, and small-scale prospective studies have demonstrated the potential link between AR signaling in the pathogenesis of these conditions. These findings position androgens and ARs as promising targets for the therapeutic management of various neurological diseases. Future placebo-controlled clinical trials are warranted to understand the efficacy and safety of androgen therapies in neurological conditions and, if appropriate, in the establishment of associated indications for use.

## Key References

- Hines M. Early androgen influences on human neural and behavioural development. Early human development 2008: 84:805–807. 10.1016/j.earlhumdev.2008.09.006.
  - A review demonstrating that early exposure to androgens influences human brain development and gender-typical behavior.
- Dengri C, Koriesh A, Babi MA, Mayberry W, Goldstein ED, Pervez M, Nouh A. Testosterone supplementation and stroke in young adults: a review of the literature. Frontiers in neurology 2024: 15:1422931. 10.3389/fneur.2024.1422931.
  - A literature review on testosterone supplementation and its potential link to stroke risk in young adults.
- Bianchi VE, Rizzi L, Bresciani E, Omeljaniuk RJ, Torsello A. Androgen Therapy in Neurodegenerative Diseases. J Endocr Soc 2020: 4: bvaa120. 10.1210/jendso/bvaa120.
  - Reviews the potential role of androgens in neurodegenerative diseases.
- Kurth F, Luders E, Sicotte NL, Gaser C, Giesser BS, Swerdloff RS, Montag MJ, Voskuhl RR, Mackenzie-Graham A. Neuroprotective effects of testosterone treatment in men with multiple sclerosis. NeuroImage: Clinical 2014: 4:454. 10.1016/j.nicl.2014.03.001.
  - An open-label phase II trial demonstrating that testosterone therapy may have neuroprotective structural brain effects in men with MS.
- Morris GL 3rd, Vanderkolk C. Human sexuality, sex hormones, and epilepsy. Epilepsy Behav 7 Suppl 2005: 2:S22–8. 10.1016/j.yebeh.2005.08.028.
  - Reviews the complex interactions between sex hormones, sexual function, and seizure activity in people with epilepsy.

## Data Availability

No datasets were generated or analysed during the current study.

## Abbreviations

DHEAS: Dehydroepiandrosterone Sulfate
DHEA: Dehydroepiandrosterone
DHT: Dihydrotestosterone
TRT: Testosterone Replacement Therapy
AR: Androgen Receptor
ASD: Autism Spectrum Disorder
ESUS: Embolic Stroke of Undetermined Source
SAH: Subarachnoid hemorrhage
MND: Motor Neuron Disease
SBMA: Spinal and Bulbar Muscular Atrophy
SOD: Superoxide Dismutase
ALS: Amyotrophic Lateral Sclerosis
DMD: Duchenne Muscular Dystrophy
DM1: Myotonic Dystrophy type 1
PD: Parkinson Disease
RRMS: Relapsing Remitting Multiple Sclerosis
MCI: Mild Cognitive Impairment
AD: Alzheimer Disease
NMDA: N-methyl-D-aspartate
GABA: Gamma-aminobutyric acid
CGRP: Calcitonin gene-related peptide
